# Physical Exploration and Diagnosis of Diseases Affecting the Respiratory Organs

**Published:** 1856-04

**Authors:** 


*Physical Exploration and Diagnosis of Diseases affecting the Respiratory Organs.* By Austin Flint, M. D.

We can only acknowledge the receipt of this work in our present number. In our next we shall endeavor to devote to it that space and careful attention which its merits demand. At present, aside from the mere announcement of its publication, we can only cordially commend it to our readers.

